# Sediment microbial fuel cells as a barrier to sulfide accumulation and their potential for sediment remediation beneath aquaculture pens

**DOI:** 10.1038/s41598-020-70002-4

**Published:** 2020-08-04

**Authors:** Christopher K. Algar, Annie Howard, Colin Ward, Gregory Wanger

**Affiliations:** 1 Department of Oceanography, Dalhousie University, Halifax, NS B3H 4R2 Canada; 2 Faculty of Engineering and Design, Carlton University, Ottawa, ON K1S 5B6 Canada; 3 Down North Technologies, Halifax, NS B3L 2Z5 Canada

**Keywords:** Biogeochemistry, Biogeochemistry, Environmental sciences, Ocean sciences

## Abstract

Sediment microbial fuel cells (SMFCs) generate electricity through the oxidation of reduced compounds, such as sulfide or organic carbon compounds, buried in anoxic sediments. The ability to remove sulfide suggests their use in the remediation of sediments impacted by point source organic matter loading, such as occurs beneath open pen aquaculture farms. However, for SMFCs to be a viable technology they must remove sulfide at a scale relevant to the environmental contamination and their impact on the sediment geochemistry as a whole must be evaluated. Here we address these issues through a laboratory microcosm experiment. Two SMFCs placed in high organic matter sediments were operated for 96 days and compared to open circuit and sediment only controls. The impact on sediment geochemistry was evaluated with microsensor profiling for oxygen, sulfide, and pH. The SMFCs had no discernable effect on oxygen profiles, however porewater sulfide was significantly lower in the sediment microcosms with functioning SMFCs than those without. Depth integrated sulfide inventories in the SMFCs were only 20% that of the controls. However, the SMFCs also lowered pH in the sediments and the consequences of this acidification on sediment geochemistry should be considered if developing SMFCs for remediation. The data presented here indicate that SMFCs have potential for the remediation of sulfidic sediments around aquaculture operations.

## Introduction

Living organisms harvest energy for their growth and metabolism by catalyzing redox reactions. While most organisms carry out both oxidation and reduction entirely within their own cells, a small subset of microbes are able to decouple these reactions, using redox shuttles and mediators, direct connections, or appendages called nanowires to access reactants outside the organism^1,2^. In some cases when microbes are provided with an electrically conductive connection across a redox gradient, they will use energy from only a single half reaction and transfer electrons to the electric circuit, capturing energy for their growth and metabolism, and producing modest amounts of electrical power as a consequence^3^. To do this, electrons generated by an oxidation reaction at an anode are transferred to a circuit either extracellularly^4,5^ or with the aid of mediators^3,6,7^, and used by another microbial population at the cathode for reduction. This allows microbes to take advantage of more favourable redox pairings than they would otherwise have access to in their immediate vicinity. Since discovery of this phenomena over 100 years ago^8^ there has been interest in harnessing it for practical applications^9^.

Devices that exploit this are termed microbial fuel cells (MFCs) and proposed uses include energy capture from wastewater treatment^9^, environmental sensors^10^, power sources for low energy devices in remote locations^11,12^ and bioremediation^13,14^. However, despite promising research and proof-of-concept protypes, they have yet to be adapted for widespread use in society, primarily due to low power generation, challenges in scaling up laboratory prototype systems, and the inability to demonstrate improvements upon current technologies^10,15^.

One application that has shown promise is sediment microbial fuel cells (SMFCs)^16^. These are MFCs that take advantage of the naturally occurring redox gradients in organic rich sediments. In sediments, diffusive transport limits oxygen supply, causing a buildup of reducing equivalents and a switch to alternative electron acceptors. These alternatives are preferentially used according to available free energy along a vertically structured redox gradient^17^ (i.e., nitrate reduction, Fe and Mn reduction, sulfate reduction, methanogenesis). By placing an electrode (anode) in the reduced layer of sediment and connecting it to a cathode in the overlying oxygenated water, an SMFC can drive current using oxygen in the water column as an electron accepter,essentially bypassing the transport limitation that gives rise to the redox gradient.

SMFCs have been tested as a means to provide energy to low power oceanographic sensors in remote settings^11,12,16,18^ and for bioremediation^14,19,20^. Recently, Kubota et al.^13^ deployed a set of five SMFCs in the organic rich sediment (12% w/w organic carbon) of Tokyo Bay, and found a reduction in porewater sulfide concentrations, identifying SMFC’s potential as a remediation technology for organic matter contaminated sediments. However, it is unclear how such an SMFC could be scaled up to combat eutrophication over an entire bay (Tokyo Bay for example has a surface area of 1,500 km^2^).

SMFCs may be useful when there are localized point sources of organic matter loading. The sediments beneath aquaculture operations may be one such environment^21,22^. These sediments receive elevated inputs of organic matter due to the deposition of unused fish feed and feces, and the spatial scale of fin-fish pens (diameters of ~ 10 m) are of a size that scaling up an SMFC might be reasonable. Gilles^23^, in a review of 65 studies of aquaculture impacts reported organic carbon concentrations beneath aquaculture cages ranging from < 1 to 26% C w/w, compared to < 1–8% C w/w at unimpacted reference sites. Such organic matter loading can alter sediment chemistry, resulting in wide ranging effects to the surrounding ecosystems and biogeochemical cycles^24^.

When organic matter builds up in sediments, sediment oxygen demand (SOD) increases and oxygen is depleted at much shallower depths^25,26^. This shoaling of the aerobic layer results in a shift to anerobic metabolism and a buildup of by-products, such as ammonium, sulfide, and methane^27^. Sulfate reduction, which produces sulfide (tot-S^2−^ = H_2_S + HS^−^ + S^2−^), can be responsible for up to 65% of total decomposition in marine sediments and increases with higher rates of organic matter loading^28^. High rates of benthic metabolism and sulfate reduction, up to an order of magnitude greater, have been observed beneath aquaculture farms relative to reference sites^27,29,30^.

Dissolved sulfide is toxic to aerobic organisms living on or in the sediments^31^. Toxicity can be observed at μmolar concentrations and effects include neurotoxicity, modification of oxygen transport proteins, and inhibition of a variety of enzymes^32^. Accumulation of sulfide can also expedite a transition to hypoxic conditions by further increasing SOD as chemoautotrophic microbes re-oxidize sulfide to obtain energy. Brooks and Mahnken^27^ reported that as sulfide concentrations increased in sediments, the number of benthic taxa declined, dropping by 50% when sulfide concentrations reached ~ 1,000 μM. The accumulation of sulfide beneath aquaculture cages is well documented^23^ and of concern, such that sediment sulfide levels form the basis of environmental monitoring regulatory regimes in many jurisdictions^33^.

**Figure 1:**
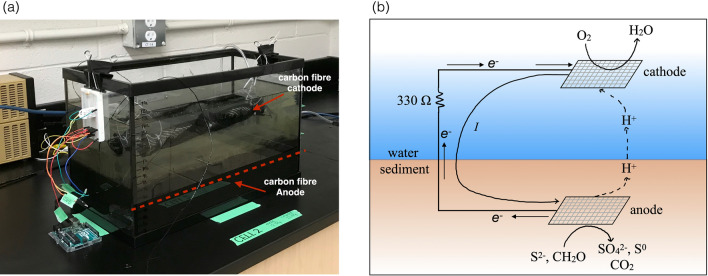
(**a**) One of the sediment microbial fuel cell microcosms used in the experiment (SMFC-2). The anode depth is indicated by the dashed red line. (**b**) Schematic diagram of the sediment microbial fuel cell showing directions of current, electron, and proton flow and possible oxidation and reduction reactions at the anode and cathode.

Evidence that SMFCs use sulfide as an electron donor^34^ and may accelerate the degradation of organic matter^35^, suggests they could be used for remediation of open-pen aquaculture sites. However, for this to be successful SMFCs must remove or prevent the accumulation of sulfide at levels comparable to those found beneath fish pens. This provides the motivation of this study, which was to quantify SMFC sulfide removal and compare this to sulfide accumulation at aquaculture sites. Our approach was to measure current generation and sulfide in laboratory SMFC microcosms over a period of 96 days. High organic matter sediments (6% C w/w)^36^ were collected from a coastal inlet and used to construct 4 laboratory microcosms in aquarium tanks. Two tanks contained operating sediment microbial fuel cells (SMFC-1 and SMFC-2) while the other two served as controls, an open circuit control (OC), and a sediment only control (SO). The open circuit control contained all the components of a sediment microbiol fuel cell, but without an electrical connection between the anode and cathode. The sediment only tank contained sediment and overlying water without microbial fuel cell components. A photograph and diagram are shown in Fig. 1a, b. To assess the influence of the SMFCs on sediment geochemistry, micro-electrodes were used to measure depth profiles of oxygen, total dissolved sulfide (tot-S^2−^ = H_2_S + HS^−^ + S^2−^), and pH at high spatial resolution (100–500 µm). Differences in profiles between the SMFCs and controls were used to determine the influence of SMFCs on sediment oxygen demand (SOD), sulfide, and pH.

## Results

### SMFC electrochemical properties

**Figure 2:**
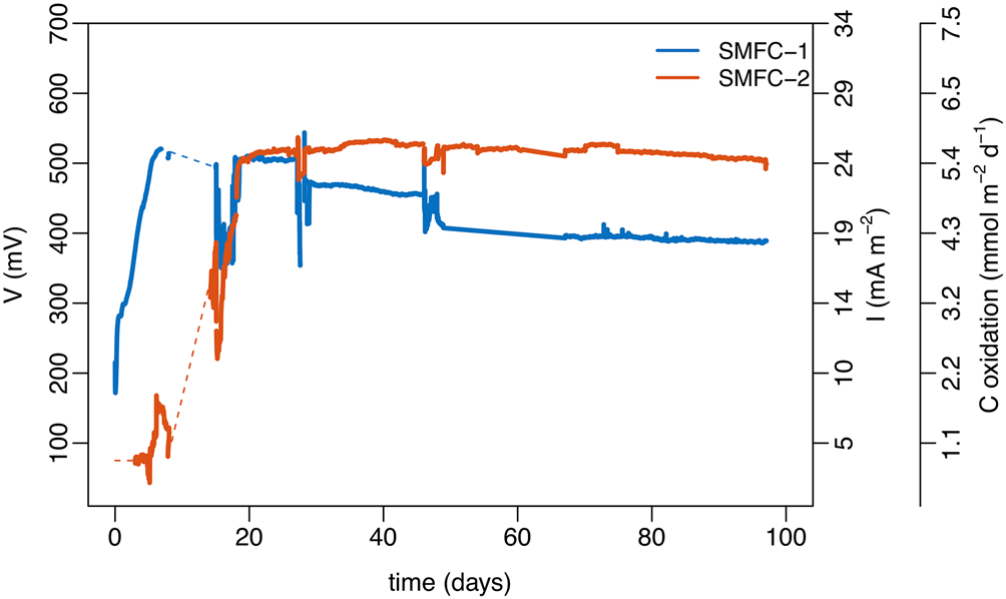
Voltage (V), and current density (*I*), recorded during the 96 day experiment for both SMFC-1 (blue line) and SMFC-2 (red line). Dashed lines show gaps in data. Current is expressed both as a current density normalized to the sediment footprint of the anode and as the equivalent rate of carbon oxidation assuming oxidation of 1 mol of carbon requires 4 mol of electrons.

Voltage and current during the 96 days of operation are shown in Fig. 2. The performance of both SMFCs were similar throughout the experiment, although SMFC-2 stabilized at a slightly higher voltage and current than SMFC-1. In both cells voltage and current were low initially, 100–200 mV and 5–10 mA m^−2^, and increased over the first 3 weeks. In SMFC-1 the highest voltage was reached on day 20 (510 mV), then decreased to 390 mV with a current density of 19.1 mA m^−2^. In SMFC-2 voltage and current density remained relatively constant at 500 mV and 24 mA m^−2^ throughout the experiment.

The electrons involved in current flow are generated through the oxidation of organic matter, though often with sulfide or other reduced substrates as intermediates. Therefore, expressing current as carbon equivalents allows a comparison with biogeochemical processes. Organic carbon oxidation can be represented by the following half reaction,

1 $$ {\text{CH}}_{2} {\text{O}} + {\text{H}}_{2} {\text{O}} \to {\text{CO}}_{2} + 4e^{ - } + 4{\text{H}}^{ + } , $$

where CH_2_O represents the stoichiometry of organic matter. Dividing current (*I*) by Faraday’s constant (*F* = 96,485 C mol^−1^) and the electrons transferred per carbon atom per turn of the half reaction (n = 4) converts current (*I*) to an oxidation rate of carbon equivalents (*C*_*ox*_),

2 $$ C_{ox} = \frac{I}{{4FA_{an} }}. $$

The rate is normalized by anode sediment footprint (*A*_*an*_) to compare with per area sediment oxygen demand (SOD) and carbon oxidation rates. This is the far-right axis of Fig. 2 and shows that current corresponds to the oxidation of 4.3 and 5.4 mmol C m^−2^ d^−1^ for SMFC-1 and 2 respectively. For comparison, this is within the range of carbon oxidation rates for coastal and continental shelf sediments in the region where the SMFC sediment was collected^37^, and represents about 38% of the background sediment oxygen demand (SOD) during the experiments, as is discussed later.

**Figure 3:**
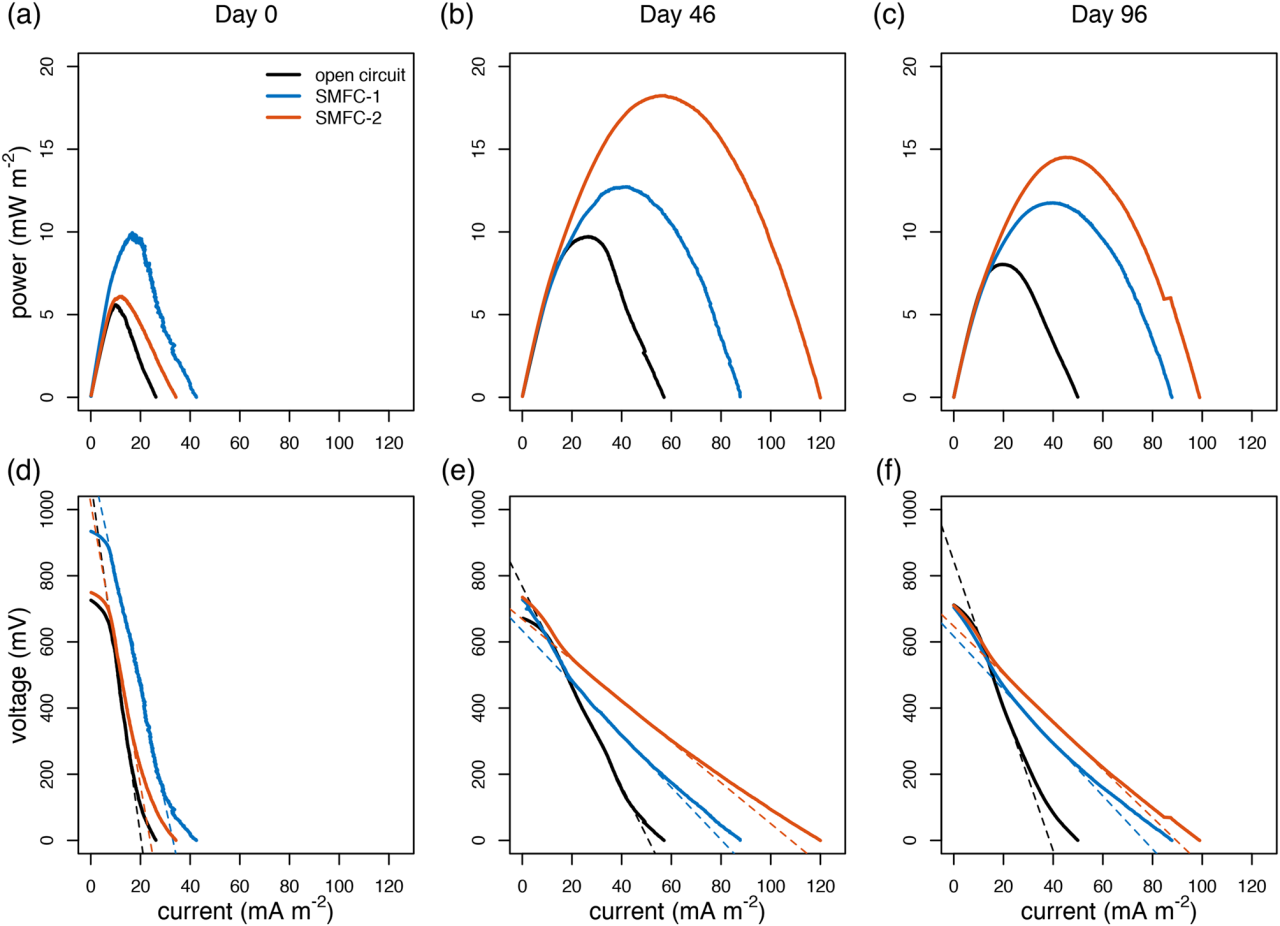
Power density, and polarization curves during the experiment for the OC (black lines), SMFC-1 (blue lines) and SMFC-2 (red lines). Power and density are shown for day 0 (**a**), day 46 (**b**), and day 96 (**c**). Polarization curves are shown for day 0 (**d**), day 46 (**e**), and day 96 (**f**). The dashed lines in (**d**–**f**) are the linear portion of the polarization curves used to determine the internal resistance.

Power density and polarization curves were used to quantify the electricity generating capability of both the SMFCs and open circuit control (OC). These are shown in Fig. 3 and Table S1 of the supplementary information. It was not determined whether the anode or cathode was the rate limiting electrode. Therefore, the curves are based on whole cell polarizations normalized to anode sediment footprint. Initially on day 0, the maximum power for the SMFCs was low (6.0–10 mW m^−2^). By day 46 it had increased to 12.7–18.2 mW m^−2^, and then declined to 11.8–14.5 mW m^−2^ by the end of the experiment (day 96). The decline in power density could be due to passivation of the electrodes^34^, decline of reactive organic matter needed to drive the system, or some combination of both. The open circuit control (OC) had lower power density than the SMFCs on all days, but its behaviour was qualitatively similar. On day 0 the power density of the OC was 5.5 mW m^−2^, on day 46 it increased to 9.7 mW m^−2^, and on day 96 it decreased to 8.0 mW m^−2^.

The internal resistance of an SMFC is the sum of ohmic losses associated with, the materials used to construct the cell, transmission losses over the wires and electrical connections, and the ability of the biofilm to transfer electrons. It can be determined from the linear portion of a polarization plot^38^ and this is indicated by the thin dashed lines in Fig. 3d–f. Internal resistance for both the OC and SMFCs declined during the first 45 days of the experiment but was consistently lower in the SMFCs. In the OC the internal resistance was 850 Ω on day 0, 240 Ω on day 46, and 347 Ω on day 96. For the SMFCs the internal resistance was 552 Ω (SMFC-1) and 671 Ω (SMFC-2) on day 0, 124 Ω (SMFC-1) and 98 Ω (SMFC-2) on day 46, and 128 Ω (SMFC-1) and 115 Ω (SMFC-2) on day 96 (Table S1).

### Microsensor profiles

**Figure 4:**
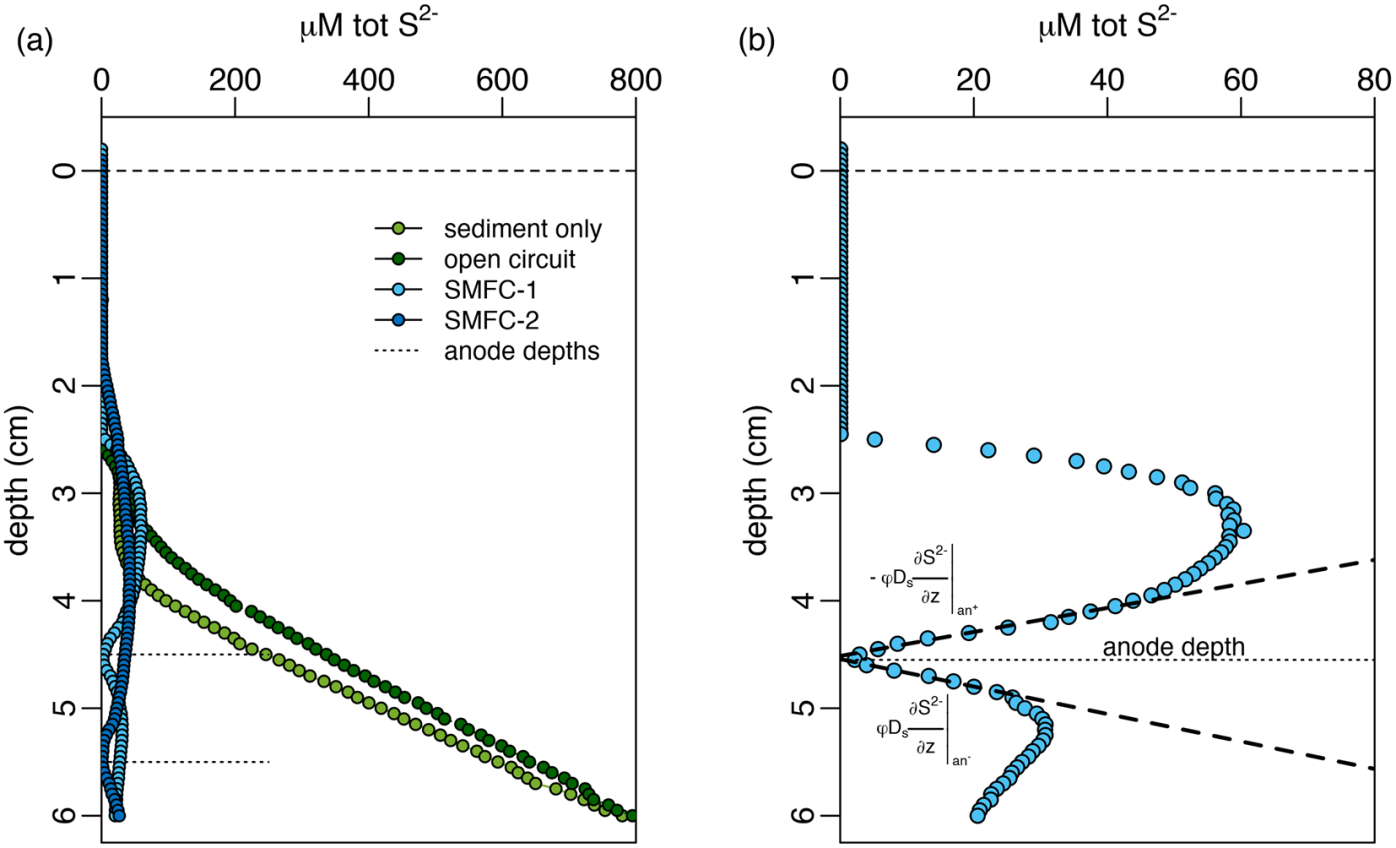
(**a**) Microsensor profiles of porewater sulfide (tot-S^2−^ = H_2_S + HS^−^ + S^2−^) for all conditions; sediment only control (SO), open circuit control (OC), SMFC-1, and SMFC-2 on day 96. Anode depths for both SMFC-1 and 2 are indicated with dotted lines and the dashed line indicates the sediment–water interface. (**b**) Sulfide depth profile for SMFC-1 on day 96 showing the drawdown of sulfide to 0 μM at the anode depth (dotted line). The heavy dashed lines above and below the anode depth is the best fit line describing the linear decrease in sulfide as the anode is approached from either above or below.

**Figure 5:**
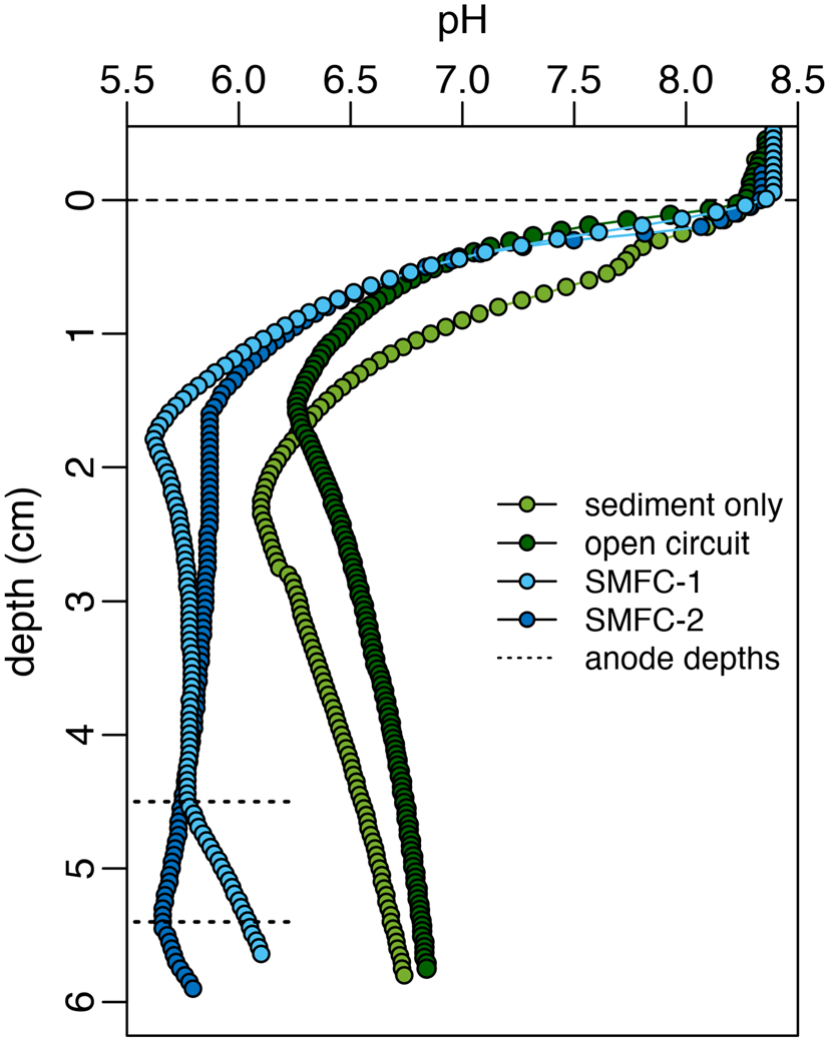
pH profiles for controls (SO and OC), SMFC-1, and SMFC-2 on day 96. The dotted lines indicate the depth of the anodes in SMFC-1 and 2 and the dashed line the sediment–water interface.

**Figure 6:**
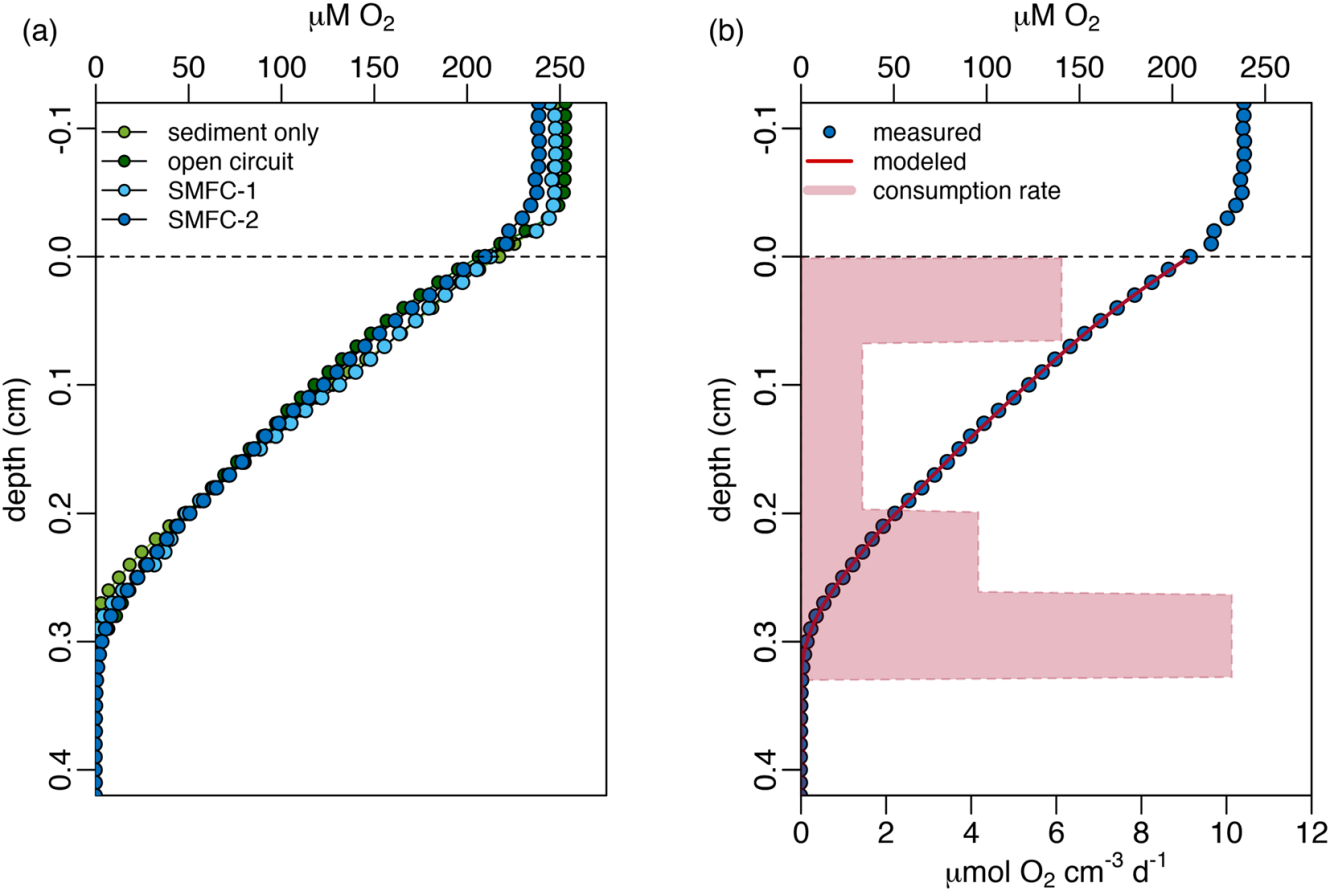
(**a**) O_2_ profiles for controls (SO and OC), SMFC-1, and SMFC-2 on day 96. The dashed line represents the sediment–water interface. (**b**) A sample profile (SMFC-2 on day 96) comparing the measured profile (blue dots) and model fit from Eq. (4) (red line). The red shaded area shows the O_2_ consumption rate needed to fit the concentration profile.

To quantify the influence of SMFCs on sediment geochemistry, microsensor profiles for oxygen, sulfide, and pH in the upper 6 cm of sediment were measured. Figures 4, 5, and 6 show representative profiles collected at the end of the experiment (day 96). Profiles at other timepoints and replicates are similar and are shown in Figures S1 and S2 of the supplementary information. Overall, the profiles indicate that functioning SMFCs had an influence on both sulfide and pH, but no measurable effect on dissolved oxygen in the porewater.

### Sulfide

Initially on day 0 there were no clear trends in the sediment sulfide profiles. Concentrations fluctuated with depth ranging from near 0 to greater than 400 μM, with no clear differences between controls and SMFCs (Figure S1 of the supplementary information). By day 46 differences between the controls (OC and SO) and SMFCs were evident, with the SMFCs having lower sulfide concentrations than the controls. This difference was further magnified at the terminus of the experiment (Figure S1 of the supplementary information). 

**Figure 7:**
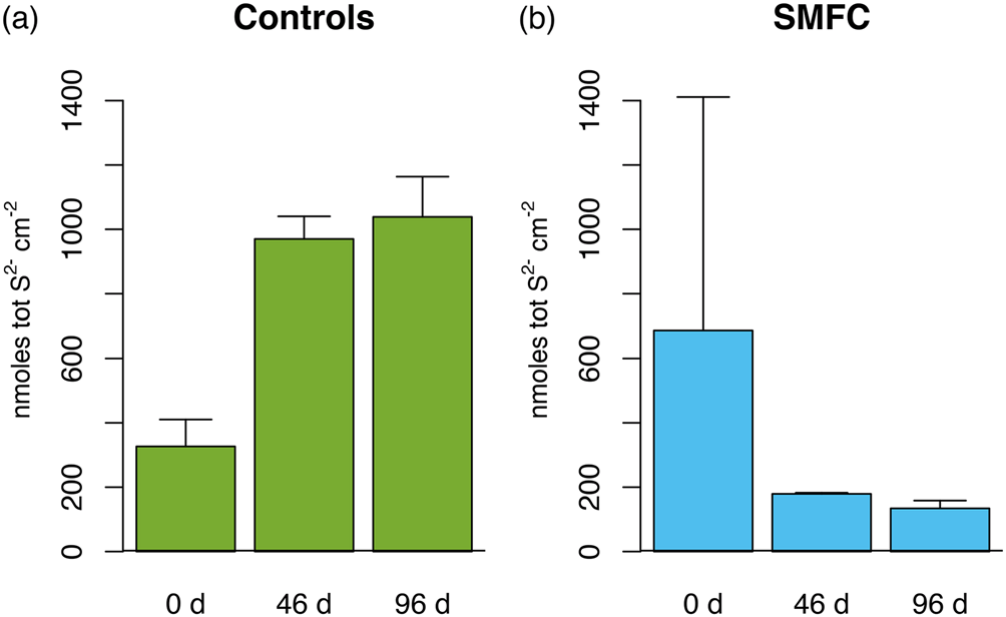
Depth integrated sulfide inventories in the top 6 cm of sediment for (**a**) controls and (**b**) SMFC.

Figure 4a compares representative sulfide profiles for the controls (SO and OC) and SMFCs on day 96. In all profiles, sulfide does not begin to accumulate until 1.5–1.75 cm depth. The spatial separation between oxygen disappearance (Fig. 6) and sulfide accumulation, the so-called sub-oxic zone, is a common feature of marine sediments and is usually due to the reaction of sulfide with dissolved iron and the formation of sulfide minerals^39,40^. Below this depth, sulfide in the OC and SO tanks reached 800 μM by 6 cm, while in the SMFCs it was less then 100 μM and dropped to 0 μM at the anode (Fig. 4b). The anode depths are indicated by the dotted lines in Fig. 4a,b. The differences in sulfide between the controls and the SMFCs is further quantified by the depth integrated sulfide inventories shown in Fig. 7. While sulfide inventories in the control tanks (OC and SO) increased during the experiment (Fig. 7a), sulfide inventories in the SMFCs decreased (Fig. 7b) and were only 20% of the SO and OC.

The low sulfide in SMFC-1, and 2, and in particular, the sharp drop in sulfide near the anode (Fig. 4b) indicates sulfide is a source of electrons to the SMFCs. By examining the gradient both above and below the anode, the flux of sulfide (tot-S^2−^) to the electrode can be determined from Fick’s law of diffusion,

3 $$ F_{an} = - \varphi D_{S}^{^{\prime}} \left. {\frac{{\partial \left[ {{\text{tot } - \text{ S}}^{{{2} - }} } \right]}}{\partial z}} \right|_{{an^{ + } }} + \varphi D_{S}^{^{\prime}} \left. {\frac{{\partial \left[ {{\text{tot } - \text{ S}}^{{{2} - }} } \right]}}{\partial z}} \right|_{{an^{ - } }} $$

where $F_{an} = - \varphi D_{S}^{^{\prime}} \left. {\frac{{\partial \left[ {{\text{tot } - \text{ S}}^{{{2} - }} } \right]}}{\partial z}} \right|_{{an^{ + } }}$ and $F_{an} = \varphi D_{S}^{^{\prime}} \left. {\frac{{\partial \left[ {{\text{tot } - \text{ S}}^{{{2} - }} } \right]}}{\partial z}} \right|_{{an^{ - } }}$ refer to the flux of sulfide (*F*_*an*_) to the anode from above and below and is estimated from the slope of the thick dashed lines in Fig. 4b. $D_{S}^{^{\prime}}$ is the tortuosity corrected diffusion coefficient^41^ and *φ* is the porosity. Table S2 in the supplementary information summarizes the results of these calculations and indicates that the flux of sulfide being oxidized by the anode for both SMFCs averaged 1.21 ± 0.65 mmol S^2−^ m^−2^ d^−1^ on day 46 and 1.75 ± 0.17 mmol S^2−^ m^−2^ d^−1^ on day 96.

### pH

Figure 5 shows the differences in pH between the control tanks (OC and SO) and SMFC-1 and 2. In the controls, pH decreased to ~ 5.5 by 1.5–2 cm depth, then gradually increased to ~ 6.5 by the bottom of the profile. In the two SMFCs, pH similarly decreased until about 1.75 cm depth, but then, rather than increase, remained low at pH ~ 5.5. The lower pH at depth in the SMFCs is due to the production of protons during the oxidation half reactions at the anode. These protons diffuse upwards, away from the anode, to counteract the flow of electrons through the external circuit.

### Oxygen

Figure 6 shows O_2_ profiles on day 96. Oxygen penetrates a few millimeters into the sediment, as is typical of organic rich coastal sediments^42^ and there appears to be no difference between the controls and SMFCs. The O_2_ penetration depth appears to deepen slightly in all four tanks from 1.5–2 mm at the beginning of the experiment to 2.5–3 mm by day 46, and 2.5–3.5 mm on day 96. Oxygen consumption rates were calculated from oxygen microsensor profiles assuming a balance between diffusive supply and microbial respiration,

4 $$ \varphi D_{{O_{2} }}^{^{\prime}} \frac{{\partial^{2} \left[ {{\text{O}}_{{2}} } \right]}}{{\partial z^{2} }} = R\left( z \right) $$

where *φ* is the porosity of the sediment, $D_{{O_{2} }}^{^{\prime}}$ is the tortuosity correct diffusion coefficient for oxygen^41^, *z* is the depth in the sediment, and *R*(*z*) is the oxygen consumption as a function of sediment depth. To solve Eq. (2) we used the approach of Berg et al.^43^. This algorithm divides *R*(*z*) into zones of constant oxygen consumption and selects the least number of zones required to fit the oxygen concentration profile. An example of this fit for SMFC-2 on day 96 is shown in Fig. 6b. The shaded red bars indicate the zones of constant oxygen consumption. Integrating these consumption rates gives the sediment oxygen demand (SOD) and the results are presented in Table S2 of the supplementary information. From these it can be seen that sediment oxygen demand (SOD) decreased in all the tanks from an average of 24.6 ± 7.9 mmol O_2_ m^−2^ d^−1^ at day 0 to 14.1 ± 3.9 mmol O_2_ m^−2^ d^−1^ by the end of the experiment (p = 0.036, one sided Welches *t* test). These values are typical of coastal sediments^42^, and measurements of oxygen uptake in Halifax Harbour near where the sediments for our experiment were obtained range between 3–30 mmol O_2_ m^−2^ d^−1^^44^. Presumably the decrease during the experiment was due to the consumption of organic matter. Comparing the SMFCs and controls did not show a significant difference in SOD, 13.1 ± 2.6 mmol O_2_ m^−2^ d^−1^ for the controls compared to 15.2 ± 5.0 mmol O_2_ m^−2^ d^−1^ for the SMFCs (*p* = 0.19, one tail Welches *t *test).

## Discussion

SMFCs provide an electrochemical connection between anoxic sediments and the oxygenated water column above, essentially functioning as a biogeochemical snorkel^45^. This allows sediment microbes to access more favorable electron acceptors (i.e. oxygen) than would otherwise be available. This suggests SMFCs could be used for the remediation of sediments impacted by point-source organic matter loading, such as the accumulation of aquaculture waste beneath fish-pens. We examined this question with a SMFC microcosm laboratory experiment that addressed two specific questions: (1) do SMFCs lower sediment oxygen demand (SOD) by accelerating organic matter decomposition? and (2) can SMFCs lower porewater sulfide concentrations to levels sufficient for the remediation of sediments beneath aquaculture farms?

To address the former, we examined changes in oxygen microsensor profiles. Since bioturbators and irrigators were absent, diffusion was the only process transporting oxygen into the sediments and depth integrated oxygen consumption rates provide a reasonable estimate of carbon remineralization (Eq. 2). If SMFCs increased organic carbon degradation, then by the end of the experiment oxygen consumption in the SMFCs microcosms should be lower than the controls. However, though consumption did decrease throughout the experiment, there was no difference between the SMFCs and controls. This suggests SMFCs did not increase the rate of organic matter remineralization. This is in contrast to Ishii et al.^35^ who found that an MFC bioreactor fed with primary clarifier effluent from a wastewater treatment plant showed complete removal of organic matter in 8–12 days, compared to 15–20 days without. However, our results are consistent with Kubota et al.^13^, who observed no change in sediment organic matter content over a 142 day deployment of SMFCs in Tokyo Bay. A likely explanation for this discrepancy is differences in organic matter reactivity and the rate limiting step for remineralization. The clarifier effluent feeding the Ishii et al.^35^ MFC reactors is likely composed of small labile organic molecules that can be easily metabolized by microbes. In this case the transport of electrons, reaction kinetics, and activity of the microbial population would control the rate of organic matter degradation. However, in sediments organic matter is composed of larger complex molecules which must first be hydrolyzed by extracellular enzymes to small labile compounds that can be more easily metabolized by microbes. It is this hydrolyzation step, determined by the reactivity of organic matter, that is often the rate limiting step^46–48^, rather than the metabolic activity of the microbial population. Therefore, unless the biofilm can significantly increase the rate of hydrolyzation of these molecules the rate of carbon remineralization would not be expected to increase.

However, although the rate of organic matter remineralization appears unaffected by the presence of the SMFCs, the distribution of remineralization between aerobic and anerobic processes is markedly different. This is indicated by the lower sulfide concentrations in the SMFCs compared to the controls (Fig. 4a). Reductions in porewater sulfide concentrations have been observed near SMFCs deployed in other marine sediments^11,12,34,49^ as well. The linear decease in sulfide at the anode (Fig. 4b) suggests sulfide is a key source of electrons for current generation. This is similar to Tender et al.^12^, who observed a linear decreases in porewater sulfide above and below an anode during an SMFC deployment in a salt marsh near Tuckerton, NJ. Likewise, Ryckelynck et al.^34^ demonstrated with a laboratory experiment, that chamber MFCs can be driven solely with sulfide as an electron donor. They proposed a mechanism for sulfur cycling near the anodes of SMFCs whereby reduced sulfur, produced from organic matter fueled sulfate reduction, is re-oxidized to provide the electrons for current generations.

However, while sulfide is clearly a source of electrons to the anode, the question remains as to the end product of oxidation and whether additional electron donors are required to balance current generation. In sediments, sulfide can be oxidized completely to sulfate (SO_4_^2−^), or partially to elemental sulfur, S^0^, according to the following half reactions,

5 $$ {\text{HS}}^{ - } \to {\text{S}}^{{0}} + 2e^{ - } + {\text{H}}^{ + } $$

6 $$ {\text{HS}}^{ - } + 4{\text{H}}_{2} {\text{O}} \to {\text{SO}}_{4}^{2 - } + 8e^{ - } + 9{\text{H}}^{ + } . $$

Whether Eq. (5) or (6) is the oxidation reaction is an important question with regards to the remediation of sulfidic sediments. If S^0^ is the end product, then its long-term fate after the SMFC is removed needs to be considered. Elemental sulfur in sediments may undergo several reactions^50^, it can be converted back to sulfide either through disproportionation^51,52^ or direct reduction and could negate some of the benefits of the SMFC. It can be converted to iron sulfides (FeS) and eventually pyrite (FeS_2_) which would immobilize it in the sediment, or ideally it can be further oxidized to sulfate^53^, decreasing the reducing capacity of the sediment as desired.

To ascertain which sulfide oxidation reaction, (5) or (6), is occurring and what additional pathways, if any, are needed for current generation, we compared the current, expressed as an electron flux, with the sulfide flux to the anode (Table S2). If reaction (5) is occurring then 2 electrons are transferred per sulfide oxidized, and sulfide reduction generates an electron flux of 3.5 mmol m^−2^ d^−1^ (day 96) to the anode, while if reaction (6) is the oxidation pathway then 14 mmol m^−2^ d^−1^ (day 96) were supplied to the anode. This could account for between 18 and 73% of the electrons required for the observed current. This suggests that other oxidation pathways in addition to sulfide oxidation are occurring at the anode, most likely the oxidation of iron sulfide minerals, or the direct oxidation of organic matter. A more detailed study of sulfur speciation and organic matter degradation in the vicinity of the anode would be needed to distinguish between these cases.

The SMFC had maximum sulfide concentrations approximately 700–750 μM lower than the controls. However, for SMFCs to be a viable tool for remediation, this difference must be comparable to the level of environmental impact. In aquaculture settings negative impacts on meiofauna communities, such as lower biomass, decreased diversity, or the invasion of opportunistic sulfide tolerant species, are observed to begin when sulfide concentrations beneath fish pens are 350–1,500 μM^31,54,55^. Recently, Cranford et al.^55^ examined a variety of benthic community health indicators and related them to sediment sulfide levels at six aquaculture farms in Canada and New Zealand. They showed that benthic ecosystem health declined dramatically when sulfide exceeded 1,000 μM and developed a scale relating ecological quality status (EQS) to sulfide in the upper sediments whereby; sulfide < 200 μM was considered “good”, above 500 μM “poor”, and > 1,000 μM “bad”, for the health of benthic communities. Based on this, the approximately 750 μM reduction in sulfide observed in our experiment would be a significant improvement to the health of benthic ecosystems impacted by aquaculture.

In addition to lower sulfide concentrations, the SMFC microcosms had lower sediment pH than the OC and SO controls. This decrease is due both to the direct generation of protons at the anode and the additional removal of alkalinity associated with the consumption of HS^−^. The effect of pH on sediment geochemistry is complicated, due to the large number of potential proton-producing and consuming reactions that may occur^56^. However, generally lowering pH will promote the dissolution of mineral phases, such as CaCO_3,_ or iron sulfides (${\text{FeS}} \to {\text{Fe}}^{2 + } + {\text{S}}^{2 - }$). The dissolution of FeS can be a positive feedback on SMFC current generation because the sulfide produced can also be oxidized by the SMFC. The liberated dissolved iron (Fe^2+^) will diffuse upwards towards the sediment surface where it is oxidized and precipitated as iron oxides. The accumulation of an iron oxide pool in the surface sediments can provide an additional layer of protection against sulfide even after the SMFC has been removed. Iron oxides have the ability to oxidize sulfides, and the Fe^2+^ produced through this sulfide oxidation can combine with additional sulfide to form FeS. While dissolved sulfide is highly toxic, FeS minerals are not. A similar iron cycling mechanism plays a role in the recently described cable bacteria “firewall” against euxinia^57^. On the other hand, FeS also binds and detoxifies heavy metals such as Cu and Zn^58^. If an SMFC induced drop in pH promoted FeS dissolution these metals could be mobilized and become bioavailable. Both Cu and Zn are additives to fish feed and Cu is a common biofouling agent applied to nets and farm equipment^59^. Although work has been done to limit their use in aquaculture^60^, the diagenetic fate of these metals due to pH changes should be considered.

A consideration of any manipulated laboratory experiment is how representative it is of the natural environment. To evaluate this, we compare current and power densities of our microcosm SMFCs to SMFCs deployed in the field. When comparing SMFC studies it is important to ensure that current and power are normalized similarly in both studies. Normalization can be done relative to the anode, cathode, or sediment footprint, depending upon the goals of the researchers. Here we only compare with studies that normalized to the anode sediment footprint as we have done. Kubota et al.^13^ obtained power densities of 11.5 mW m^−2^ and current densities between 10 and 30 mA m^−2^ for SMFCs deployed in Tokyo Bay. Tender et al.^12^ and Ryckelynck et al.^34^ report power densities of 10–30 mW m^−2^ from SMFCs deployed in a salt marsh^2^, and 30 mA m^−2^ and 11 mW m^−2^ for an SMFC in a coastal estuary. The similarity in power and current densities between the field deployed SMFCs and ours gives confidence that our results are transferrable to the natural environment.

Interestingly, the electrogenic properties (power density and internal resistance) of the OC improved between day 0 and 46. This suggests the mere presence of the carbon fiber electrode might provide a surface for the development of a biofilm with some potential for electron transfer regardless of whether it had been connected to a circuit or not. Sediments have steep redox gradients and one possible explanation is that a conductive material placed in the sediments could encourage biofilm formation by providing electrical connections across small scale redox gradients. However, the higher internal resistance of the OC (247–347 Ω) compared to the SMFCs (98–128 Ω) suggests that any biofilm that developed on the OC electrode was less adapted to carry out this task. SMFC-1 and 2 had selective pressure, in the form of a voltage gradient, to shift the biofilm community toward those members of the microbial community that could take advantage of the electrical connection to the overlying water while the OC did not.

In conclusion, we have demonstrated that SMFCs have the potential to remove sulfide from sediments at a scale that could benefit the benthic environment beneath aquaculture pens. Aquaculture is a growing industry and its expansion is only expected to accelerate as it becomes increasingly difficult for wild-capture fisheries to meet society’s demand for marine protein^61^. Therefore, solutions to environmental problems, such as organic matter loading and sulfide accumulation, need to be developed. SMFCs are a promising solution, provided they can be scaled up. Most SMFCs deployed in the environment generally are on the order of 0.5 m^2^ ^11–13,34,49^, still much smaller than aquaculture fish pens which have diameters of 10 s of meters. Nevertheless, although there are likely challenges to be overcome, this work will inspire future research aimed at investigating the feasibility of SMFCs as a solution to this persistent environmental problem.

## Methods

### Experimental setup

Ten sediment cores (9.5 cm ID × 60 cm long) of organic rich (6% C w/w), fine-grained sediment^36^ were collected from the Northwest Arm, Halifax NS, Canada (44.6313 N, − 63.5960 W) on July 20th, 2018 using a KC Denmark multi-corer. Sediment was transferred to coolers for transport to Dalhousie University and used to set up 4 laboratory scale sediment microbial fuel cells (SMFC). Sediment was homogenized and 8L was added to four 20.8L aquarium tanks (40.6 cm × 20.5 cm × 25 cm) and allowed to settle overnight. The next day carbon fiber anodes were placed on the sediment surface in three of the tanks, buried with an additional 2L of sediment and 8.5L of Northwest Arm seawater was carefully siphoned into each tank so as to minimize the disturbance of the sediment surface.

A different treatment was assigned to each of the tanks. Three of the tanks contained microbial fuel cell components; an anode and cathode. In two of these the anode and cathode were connected with a 330 Ω resistor (SMFC-1 and SMFC-2), while in the third the anode and cathode were not connected but left in an open circuit configuration (OC). The fourth tank contained only sediment (SO) and served as a control. The microbial fuel cells were operated for a period of 96 days. Experiments were conducted at the ambient temperature of the laboratory, 21 °C.

### Electrode construction

For each anode, five rows of titanium wire were woven lengthwise, 2.5 cm apart into 35 × 18 cm pieces of carbon fabric (Composites Canada twill weave carbon fabric, Part number: CA058TW-50), which was flame treated to remove any pre-existing coatings. We have previously observed that flame treatment decreases the internal resistance of the carbon fiber and improves microbial fuel cell performance (*G. Wanger unpublished data*)*.* The wire was attached to an external circuit containing the 330 Ω resistor.

For each cathode, titanium wires were woven 5 cm apart into a 29 × 41 cm flamed carbon fabric sheet. The carbon sheets were then wrapped around an air stone 25 cm in length, to ensure oxygen concentrations remained near saturation at the cathode.

### Data collection

Throughout the experiment the voltage drop across the 330 Ω resistors in SMFC-1 and 2 was continuously logged at 10 min intervals using an Arduino Uno connected to a Raspberry Pi computer. The Arduino based voltage logger was calibrated against a handheld multimeter prior to the beginning of the experiment and agreement was shown to be better than 2%. Voltages were regularly checked against the handheld multimeter throughout the experiment. On days 0, 46, 96, more detailed measurements were made. This included cell polarization measurements for each SMFC and the OC, and high-resolution depth profiles for porewater O_2_, pH, and sulfide over the top 6 cm of sediment in each tank. A depth that includes sediment both above and below the anode.

Whole cell polarization measurements were determined using a Gamry Reference 600 + Potentiostat in a two electrode configuration. During measurements, voltage was swept across a 700 mV range beginning at open circuit potential and decreasing to 0 at a scan rate of 1 mV s^−1^. Power output at each voltage can be calculated from Ohm’s Law (*P* = *VI*) and the internal resistance of each cell was determined from the linear portion of *I* versus *V* curves^38^. Polarization measurements were not performed for the anode and cathode individually, and no attempt was made to determine which electrode was rate limiting. All polarization results refer to whole cell potentials and were normalized to the anode sediment footprint.

Depth profiles of porewater sulfide (tot-S^2−^ = H_2_S + HS^−^ + S^2−^), pH, and O_2_ were measured using a Unisense microprofiling system. Oxygen concentrations were determined in the top 0.5 cm of sediment at 100 μm increments using a Clark-type electrode with a 100 μm tip (Unisense OX-100). pH and H_2_S were measured to 6 cm depth; this was deeper than the depth anodes were buried in the sediment. The microsensor tips were small enough to easily penetrate through the anode and therefore could measure concentrations above, below, and at the anode surface. pH was measured with a Unisense pH-100 electrode every 500 μm down to 6 cm. H_2_S measurements were made at the same resolution as pH using a 100 μm tip Unisense Type I SULF sensor. This measures current generated when H_2_S is oxidized directly at the sensor anode and can be converted to a measurement of total dissolved sulfide (tot-S^2−^ = H_2_S + HS^−^ + S^2−^) using the equations of Millero et al.^62^ provided pH has been measured. To minimize disruption to the sediment redox gradient, only single profiles for oxygen, pH, and sulfide were taken on days 0 and 46. This is because profiling to 6 cm depth results in a hole several mm in diameter in the sediment which could potentially provide a pathway for oxygen to reach deeper sediment layers, disrupting the redox gradient driving the SMFC. On the final timepoint (day 96) three profiles for each solute were made at different locations within each tank to assess spatial variability. Profiling locations in the tanks were randomly chosen but were at least 5 cm away from the walls of the tanks to minimize edge effects.

## Supplementary information

Supplementary Information.

## Data Availability

The data sets are available from the corresponding author on reasonable request.
