# Supplementary material for: Sediment microbial fuel cells as a barrier to sulfide accumulation and their potential for sediment remediation beneath aquaculture pens
Source: Sci Rep. 2020 Aug 4;10:13087. doi: 10.1038/s41598-020-70002-4 (PMC7403589; doi:10.1038/s41598-020-70002-4)
Supplement: Supplementary file 1 — Supplementary Information. [file 41598_2020_70002_MOESM1_ESM.pdf]

Supplementary Information

Sediment microbial fuel cells as a barrier to sulfide accumulation and their potential for sediment remediation beneath aquaculture pens

Christopher K. Algar*[†], Annie Howard[†], Colin Ward[‡], and Greg Wanger[§]

[†]Dept. of Oceanography, Dalhousie University, Halifax NS. B3H 4R2

[‡]Faculty of Engineering and Design, Carlton University, Ottawa ON. K1S 5B6

[§]DownNorth Technologies, Halifax, NS. B3K 2Z8

*Corresponding author: Christopher Algar, chris.algar@dal.ca, Dept of Oceanography, Dalhousie University, Halifax, NS. CANADA.

Table S1: Polarization and power characteristic for sediment microbial fuel cells (SMFC-1 and 2) and the open circuit (OC) condition.

	**max. power ($mW\ m^{-2}$)**	**current at max. power ($mA\ m^{-2}$)**	**max. current ($mA\ m^{-2}$)**	**internal resistance (Ω)**
**OC**				
0 d	5.5	10.0	26.2	850
46 d	9.7	26.5	57.1	240
96 d	8.0	19.5	49.9	347
**SMFC-1**				
0 d	10.0	16.7	42.5	552
46 d	12.7	42.2	87.7	124
96 d	11.8	39.8	87.8	128
**SMFC-2**				
0 d	6.1	12.2	34.3	671
46 d	18.2	57.4	120.0	98
96 d	14.5	44.9	99.0	115

Table S2: Activity and fluxes of O_2, tot-S^{2-} and electrons during the SMFC experiment. Electron flux was determined by dividing the current density by Faraday's constant. (96485 C mol^{-1}). For the No SMFC avg, both the sediment only (SO) and open circuit (OC) conditions were averaged and SMFC-1 and 2 were averaged for the SMFC avg case. The electron flux and tot-S^{2-} flux to anode are only relevant for the conditions with operating SMFCs.

	O_2 consumption (mmole m^{-2} d^{-1})			electron flux (mmole e^- m^{-2} d^{-1})			tot-S^{2-} flux to anode (mmole m^{-2} d^{-1})		
	0d	46d	96d	0d	46d	96d	0d	46d	96d
Control	21.1	17.9	17.1 ± 6.1	-	-	-	-	-	-
OC	32.0	15.4	13.2±3.6	-	-	-	-	-	-
SMFC-1	30.1	30.1	13.5±2.2	11.02±1.67	18.89±0.87	16.69±0.05	-	0.75	1.83±0.18
SMFC-2	15.0	30.1	12.7±3.4	3.28±0.12	22.17±0.57	21.70±0.10	-	1.67	1.67±0.14
No SMFC avg.	26.6 ± 7.7	16.6 ± 1.7	15.2 ± 5.0	-	-	-	-	-	-
SMFC avg.	22.6 ± 10.6	25.0 ± 7.2	13.1±2.6	7.1±5.5	20.53±2.32	19.20±3.54	-	1.21±0.65	1.75±0.17
All tanks avg.	24.6 ± 7.9	20.8 ± 6.4	14.1 ± 3.9	-	-	-	-	-	-

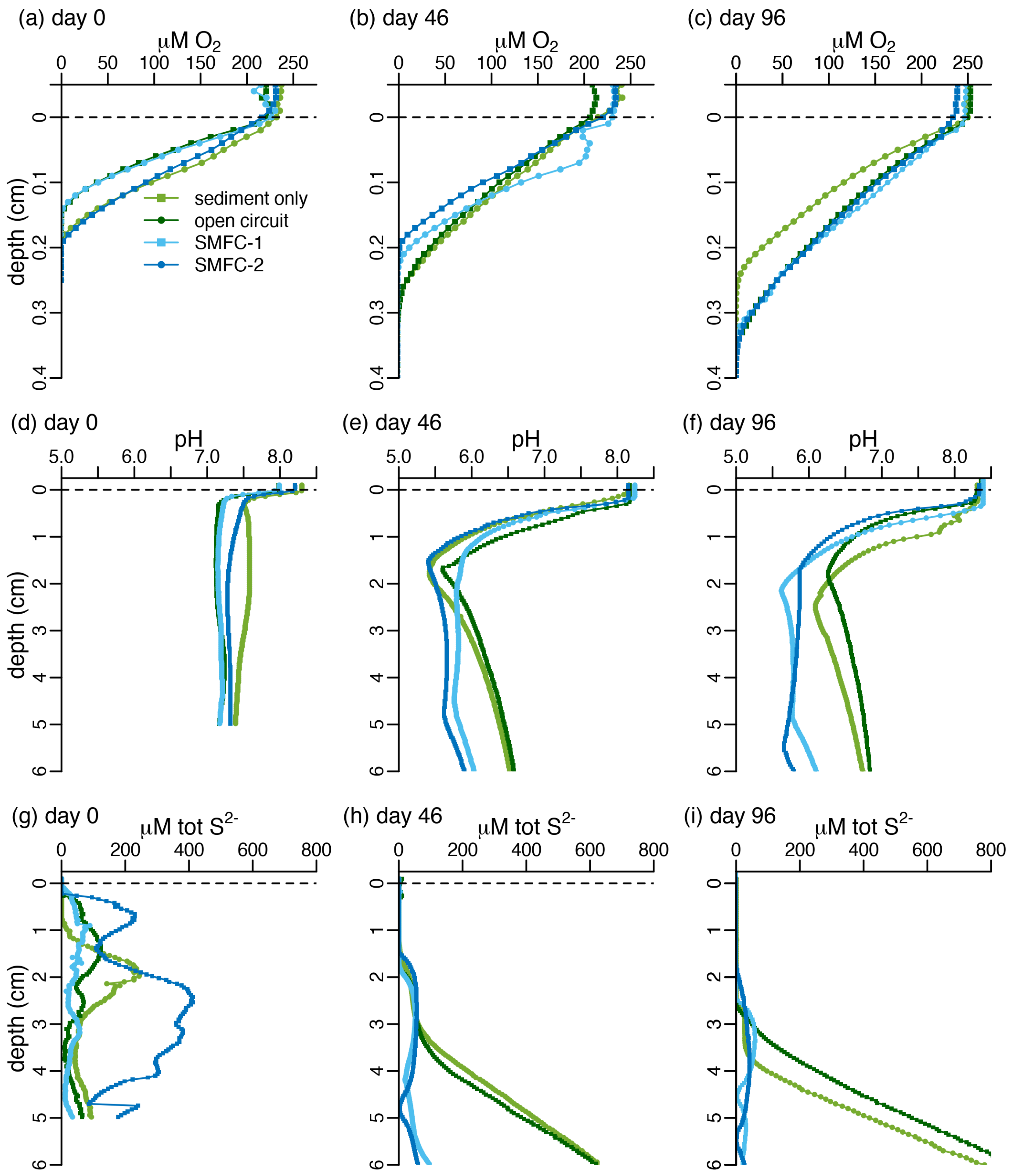

Figure S1: Microsensor profiles for O_2 (a,b,c), pH (d,e,f), and tot-S^{2-} (g,h,i) for SMFC-1 (light blue squares), sediment only (light green, squares), open circuit (dark green, circles), SMFC-1 (light blue, squares), and SMFC-2 (dark blue, squares). On days 0, 46, and 96. Only a single representative profile is shown for oxygen, pH, and tot-S^{2-} for day 96.

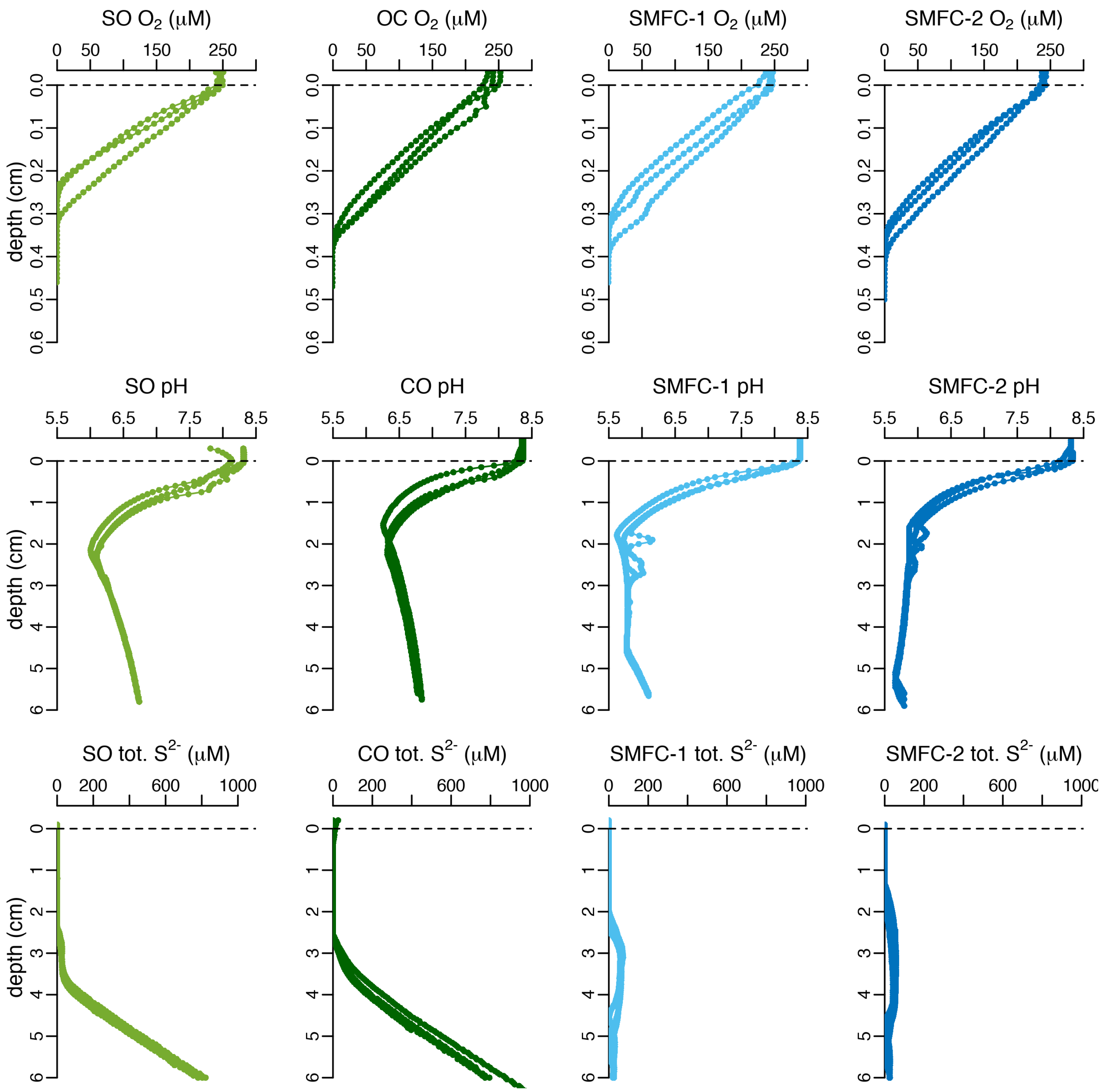

Figure S2: Spatial variability of microsensor profiles in each tank for oxygen (top row), pH (middle row), and total sulfide (bottom row) at the end of the experiment (day 96). Three profiles for each solute were made at random locations in each tank. Sediment only (SO) control - light green, open circuit; (OC) control – dark green; SMFC-1 – light blue; SMFC-2 – dark blue.
